# Pre-treatment serum vascular endothelial growth factor is associated with clinical response and overall survival in advanced melanoma patients treated with ipilimumab

**DOI:** 10.1186/2051-1426-1-S1-P247

**Published:** 2013-11-07

**Authors:** Jianda Yuan, Jun Zhou, Zhiwan Dong, Sapna Tandon, Deborah Kuk, Katherine S Panageas, Philip Wong, Jedd D Wolchok, F Stephen Hodi

**Affiliations:** 1 Ludwig Center for Cancer Immunotherapy, Memorial Sloan-Kettering Cancer Center, New York, NY, USA; 2 Department of Medicine, Memorial Sloan-Kettering Cancer Center, New York, NY, USA; 3 Department of Epidemiology and Biostatistic, Memorial Sloan-Kettering Cancer Center, New York, NY, USA; 4 Weill Cornell Medical College of Cornell University, New York, NY, USA; 5 Department of Medical Oncology, Center for Immuno-oncology, Dana-Farber Cancer Institute and Harvard Medical School, Boston, MA, USA

## 

Ipilimumab, an antibody that blocks cytotoxic T lymphocyte antigen 4 (CTLA-4), had shown improved overall survival (OS) for patients with metastatic melanoma. However predictive biomarkers for clinical benefit have not been well defined. We aimed to evaluate serum vascular endothelial growth factor (VEGF) and its association with clinical benefit and OS for ipilimumab treated advanced melanoma patients. Sera were collected from 176 patients treated with ipilimumab at 3 (n=98) or 10 mg/kg (n=68) from 2005 to 2013. We analyzed serum VEGF at baseline and at the end of induction (week 12) by Meso Scale Discovery kit. The association VEGF with clinical benefit and OS was analyzed using Fisher's exact test and Kaplan-Meier log-rank test. Pre-treatment VEGF value correlated with clinical benefit for 157 melanoma patients with the availability of clinical response at wk24 (p=0.0111) using 43 pg/ml as the cutoff of baseline VEGF value defined by maximally selected log-rank statistics. High level of soluble pre-therapy VEGF (≥ 43 pg/ml) in blood was associated with decreased OS, as compared to low level baseline VEGF ( < 43 pg/ml) (Median OS 6.6 vs 12.9 months , p=0.006 for all 176 patients; median OS 7.4 vs 14.3 months, p=0.037 for 3 mg/kg group; median OS 6.2 vs 10.9 months, p=0.048 for 10 mg/kg group, respectively). High level of soluble VEGF at wk12 was correlated with OS in all patients as well (p=0.023). There was no correlation between the change of VEGF and clinical outcome. Serum VEGF may be a predictive biomarker to ipilimumab treatment, and prospective investigation warranted.

**Figure 1:**
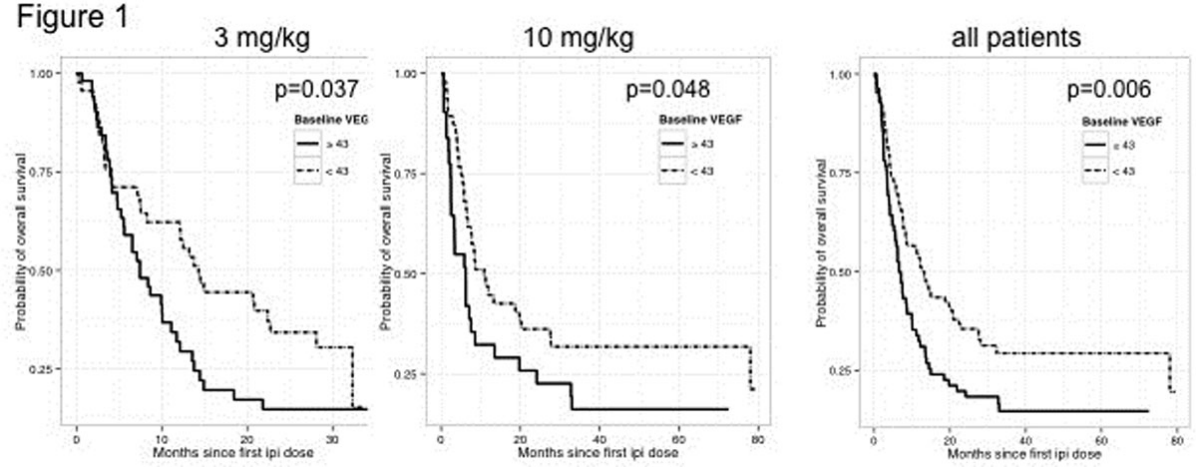
Kaplan Meier curve of overall survival by using 43 pg/ml as the cutoff for baseline VEGF

